# *Piscine orthoreovirus* infection in Atlantic salmon (*Salmo salar*) protects against subsequent challenge with infectious hematopoietic necrosis virus (IHNV)

**DOI:** 10.1186/s13567-018-0524-z

**Published:** 2018-03-13

**Authors:** Niccoló Vendramin, Anna Luiza Farias Alencar, Tine Moesgaard Iburg, Maria Krudtaa Dahle, Øystein Wessel, Anne Berit Olsen, Espen Rimstad, Niels Jørgen Olesen

**Affiliations:** 1 National Veterinary Institute, Technical University of Denmark, Copenhagen, Denmark; 2 Norwegian Veterinary Institute, Oslo, Bergen, Norway; 3 Department of Food Safety and Infection Biology, Norwegian University of Life Sciences, Oslo, Norway

## Abstract

Infectious hematopoietic necrosis virus (IHNV) is endemic in farmed rainbow trout in continental Europe and in various salmonid fish species at the Pacific coast of North America. IHN has never occurred in European Atlantic salmon (*Salmo salar*) farms, but is considered as a major threat for the European salmon industry. Another virus, *Piscine orthoreovirus* (PRV), is widespread in the sea phase of Atlantic salmon, and is identified as the causative agent of heart and skeletal muscle inflammation. The aim of this study was to investigate the interactions between a primary PRV infection and a secondary IHNV infection under experimental conditions. A PRV cohabitation challenge was performed with Atlantic salmon. At peak of PRV viremia the fish were challenged by immersion with an IHNV genogroup E isolate. Clinical signs and morbidity were monitored. Target organs were sampled at selected time points to assess viral loads of both pathogens. Antiviral immune response and presence of histopathological findings were also investigated. Whereas the PRV-negative/IHNV positive group suffered significant decrease in survival caused by IHNV, the PRV infected groups did not suffer any morbidity and showed negligible levels of IHNV infection. Antiviral response genes were induced, as measured in spleen samples, from PRV infected fish prior to IHNV challenge. In conclusion, PRV-infection protects Atlantic salmon against IHNV infection and morbidity, most likely by inducing a protective innate antiviral response.

**Electronic supplementary material:**

The online version of this article (10.1186/s13567-018-0524-z) contains supplementary material, which is available to authorized users.

## Introduction

Viral pathogens constantly challenge finfish aquaculture. In net pens, farmed stocks can be exposed to pathogens through water at any given time. Therefore, knowledge about effects of co-infections and pathogen interactions is important for development and implementation of effective disease control strategies.

Infectious hematopoietic necrosis virus (IHNV) is a member of the *Rhabdoviridae* family, genus *Novirhabdovirus*, which are bullet shaped viruses with non-segmented, negative single stranded RNA genome. IHNV is the causative agent of infectious hematopoietic necrosis (IHN), a widespread disease mainly found in salmonid fish species in western North-America, continental Europe and Asia [1]; IHN belongs to the list of notifiable listed disease according to current legislation [2]. Clinically affected fish externally show the skin darkening, exophthalmia and pale gills. Common necropsy findings are pale internal organs with petechial hemorrhages, and intestines often filled with mucus-like fluid [3].

Phylogenetic analyses of the genetically diverse G gene of IHNV define five major genogroups (U, M, L, E, J), which broadly refer to the geographical distribution of the genogroups [3, 4].

The presence of IHNV in Europe was first confirmed in 1987, with at least two different introductions in Italy and France, with viruses originating from the M genogroup. Since then, IHNV has spread in different European countries, evolving separately and constituting the E genogroup which is the youngest within IHNV genogroups [5]. IHNV is currently endemic in continental Europe and can be associated with significant losses in freshwater farmed rainbow trout (*Onchorynchus mykiss*), while countries producing Atlantic salmon in northern Europe are declared officially free from the virus according to European legislation [2]. The virus is widespread in western North-America, including seawater areas with Atlantic salmon farming, where a DNA vaccine is used to control the disease [6]. In the seawater phase both Pacific salmonid species and Atlantic salmon are susceptible to infection [7].

*Piscine orthoreovirus* (PRV) is ubiquitous in farmed salmon in Norway during the sea water phase, and has emerged in recent years as a relevant threat for Atlantic salmon aquaculture being the etiological agent of heart and skeletal muscle inflammation (HSMI) [8–12]. PRV is a non-enveloped virus with a segmented double-stranded RNA genome enclosed in a capsid with two concentric protein layers [13], currently PRV cannot be cultivated in vitro un cell culture monolayers. The gross pathological findings of HSMI point towards circulatory failure, and characteristic histopathological findings are epi-, endo- and myocarditis, myocardial necrosis, red skeletal myositis and necrosis [14]. PRV infection in Atlantic salmon induces a strong innate antiviral immune response in its major target cell, the erythrocyte [15], and thus this response can be measured in any vascularized organ, and has been described in various organs such as spleen, head kidney and heart tissue [10, 16].

In this study, we show that a preceding PRV infection in Atlantic salmon interferes with a subsequent challenge with genogroup E IHNV, fully protecting the fish from infection and IHN disease development. The experiment also demonstrated the susceptibility of Atlantic salmon for IHNV, genogroup E.

## Materials and methods

### Experimental design and fish sampling

The experiments were carried out in the facilities at DTU-VET (Frederiksberg, Denmark) in accordance with the recommendations in the current animal welfare regulations under the license 2013-15-2934-00976. The protocols were approved by the Danish Animal Research Authority.

The fish were monitored on a daily basis regarding state of health and environment.

Atlantic salmon juveniles (mean weight of 5 g) were imported from a commercial farm certified free from listed diseases according to EU legislation (CD 2006/88) also including infectious pancreatic necrosis virus (IPNV) and bacterial kidney disease (BKD). The fish were brought into the quarantine facility, using recirculated tap water disinfected by UV, temperature = 12 °C ± 1, and kept there for 75 days. Before starting the trial, the experimental fish were screened for IHNV, IPNV and viral haemorrhagic septicemia virus (VHSV) inoculating organ homogenate on cell culture according to a Council implementing decision 2015-1554 [17]. Furthermore, fish were screened by qPCR for salmonid alphaviruses (SAV), according to OIE Manual of Diagnostic Tests for Aquatic Animals [18] and PRV [9]. Bacteriological analysis was performed according to standard diagnostic procedures, by streaking kidney tissue onto blood agar (BA) and Tryptone Yeast Extract Salts (TYES), followed by incubation for 1 week at 20 and 15 °C, respectively. All tests for all pathogens were negative prior to the infection trial.

**Figure 1:**
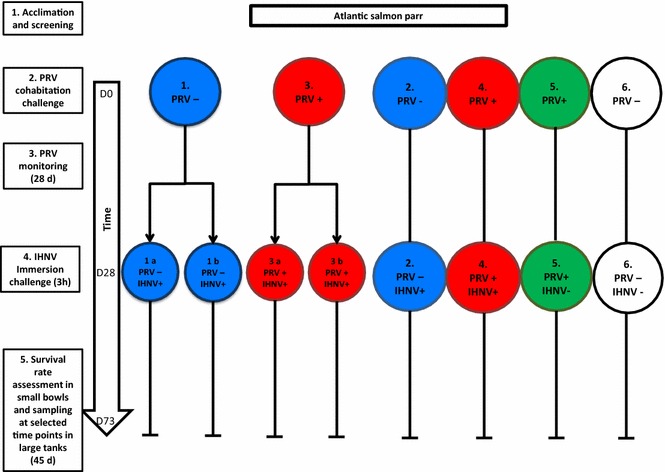
**Experimental design.** After acclimation and health screening (1), Atlantic salmon parr were divided in 6 × 150 L tanks and PRV cohabitation challenge was performed. Each tank contained each 40 shedders or mock-shedders (controls) and 55 cohabitants (2). After monitoring PRV infection for 4 weeks (3), IHNV bath challenge was performed (4). At this stage 32 shedders and 47 cohabitants were present in each tanks. The trial continued for 45 days after IHNV trial. (5) Sampling at selected time points continued in large tanks, while pathogenicity assessment was conducted in duplicate small 8 L bowls (10 shedders and 10 cohabitants) (tanks 1a, b and 3a, b).

An overview of the experimental design is displayed in Figure 1. After the quarantine period and health screening, 570 Atlantic salmon (*Salmo salar L.*) (mean weight 15 g) were transferred to the experimental facilities at DTU-VET and separated into six tanks (150 L tanks (Table 1) run with 15 L/h flow-through fresh water renewal at the following conditions: 12 °C ± 1 °C, L:D 12:12, stocking density below 70 kg/m^3^, and feeding of 1.5% of biomass.

**Table 1:** Prevalence of hearts with lesions consistent with HSMI

Treatment	Shedders	Cohabitants	Total
	Positive/n	Positive/n	Positive/n
Negative control	–	–	0/17
PRV−/IHNV+	–	–	0/21
PRV+/IHNV−	16/20	16/21	32/41 (78%)
PRV+/IHNV+	18/21	14/21	32/42 (76%)

For the pathogenesis study the 150 L tanks of each group PRV−/IHNV+, PRV+/IHNV+, PRV−/IHNV−, and PRV+/IHNV− were repeatedly sampled to monitor virus kinetics and antiviral responses. To study pathogenicity, duplicate tanks of the groups PRV−/IHNV+ and PRV+/IHNV+ (20 fish per group) were transferred to small bowls (8 L) upon IHNV challenge and monitored for clinical signs, disease development and mortality for 45 days.

Prior to organ sampling, fish were anesthetized with benzocaine chloride (800 mg/1 L water) and euthanized by cervical transection. During the first 4 weeks post PRV challenge, blood, spleen and heart samples were taken weekly from two shedders and two cohabitants from PRV exposed and non-exposed tanks (tanks 3–6). Weight and length of each animal were recorded.

In the second part of the trial, i.e. after the IHNV challenge, three shedders and three cohabitants from each of the 150 L tanks (2, 4, 5 and 6) were sampled more frequently, i.e. at 2, 5, 12, 19, 26 and 33 days post IHNV infection. During this latter phase, head kidney was added to the sampling sets. Spleen and head kidney were stored in RNALater^®^ (ThermoFisher Scientific Inc, USA). Hearts were divided along the midsagittal line, storing one part in RNALater^®^ (ThermoFisher Scientific Inc) and the other in 10% buffered formalin for histopathological assessment. Blood was sampled from the caudal vein on heparinized tubes (BD Biosciences, USA) and aliquoted for both hematocrit and hemoglobin evaluation in a Hematology Analyzer (Vet ABC, Scil, USA) and to prepare blood smears for immunofluorescence staining. The experiment was terminated 45 days after IHNV challenge (corresponding to 73 days after PRV challenge).

### Challenge trials

#### PRV cohabitation challenge

The PRV inoculum used for infection originated from a field outbreak of HSMI in Norway in 2012 that had subsequently been passaged in three PRV challenge experiments at VESO Vikan (Oslo, Norway) in which HSMI was reproduced [19]. Briefly, heparinized blood was collected from three PRV infected fish 7 weeks post cohabitation challenge. Blood was centrifuged to remove plasma and the remaining blood pellet was pooled and diluted 1:3 in Leibovitz’s L15 medium (Life Technologies, USA) supplemented with gentamicin at 50 μg/mL (Gibco, USA) and Fungizone at 0.25 g/mL (Gibco). The pellet kept on ice was sonicated ten times for 10 s each at 20 kHz and centrifuged at 2000 ×* g*. The resulting supernatant was shown to contain high loads of PRV as determined by RT-qPCR (Ct value of 18.6/5 µL inoculum).

Fish dedicated to be shedders (*n* = 40 per tank) were injected intra-peritoneal (i.p.) with 0.1 mL of challenge material under anesthesia with benzocaine (80 mg/L) and marked by clipping of the adipose fin. Fifty-five naïve unmarked cohabitants were added to each tank, resulting in a shedder ratio of 42%.

In PRV negative tanks shedders were mock injected with 0.1 mL of naïve rainbow trout blood (tested negative for PRV by RT-qPCR) prepared like the PRV infected inoculum.

#### IHNV immersion challenge

Four weeks post PRV infection, upon the peak of PRV viremia (median PRV Ct value obtained in cohabitants = 19.8), IHNV challenge was performed in tanks 1, 2, 3 and 4. Before IHNV challenge 40 fish from each of the duplicate tanks 1 and 3 (PRV−/IHNV+ and PRV+/IHNV+) were transferred to 8 L bowls (20 fish in each, i.e. 10 PRV shedders and 10 PRV cohabitants). In order to maintain the same biomass during the challenge time (3 h) fish from tanks 2 and 4 were transferred into 30 L tanks for the IHNV challenge, and then transferred back in the original 150 L tanks. Remaining fish from tank 1 and 3 were excluded from the trial.

For IHNV challenge, the German isolate DF04/99, proven to be highly virulent to rainbow trout [20] was propagated in EPC (*Epithelioma Papulosum Cyprini*, ATCC^®^ C RL-2872™) cell monolayers at 15 °C, frozen once and titrated (Kärber method) [21] in EPC cells. IHNV immersion challenge dose was estimated as 10^6^ TCID_50_/mL, whereas fish from tank 5 and 6 where mock challenged with sterile L15 media. Fish were challenged for a period of 3 h.

Fish separated into the bowls for the pathogenicity study were monitored daily for clinical signs of disease. Individual fish displaying clinical signs of IHN (apathy, skin darkening, exophthalmos and abnormal behavior) were euthanized by an overdose of benzocaine chloride (500 mg/L). Head kidney, heart and spleen from clinically affected fish were sampled and pooled in MEM (Minimum Essential Medium, Sigma Aldrich, USA) following the criteria of one pool per bowl per day, for later confirmation of IHNV infection by RT-qPCR.

### RT-qPCR for assessing viral loads

RT-qPCR was performed on RNA purified from spleen. Total RNA was purified using Qiagen RNeasy mini kit (QIAGEN, Germany) and the final RNA eluted in 30 µL of RNAse free water. RNAse free water was used as a negative control and positive blood sample was used as a positive control.

RT-qPCR was carried out using QuantiTect Probe RT-PCR Kit (QIAGEN), according to the manufacturer’s instructions. Briefly, for each sample, 5 µL of purified RNA was mixed with 4.75 µL of RNAse free water; 12.5 µL of 2X QIAGEN Quantitect Probe Mix; 1 µL of each primer at 10 µM; 0.5 µL of the probe and 0.25 µL of QIAGEN Quantitect Enzyme Mix. RNAse free water was used as a negative control. Primers, probes and working conditions for RT-qPCR for PRV and IHNV are described elsewhere [9, 22].

RT-qPCR was performed on Stratagene Mx3005P and Mx3000P qPCR-systems, with MxPro (v. 4.10) software used for RT-qPCR data analysis. According to internal Standard Operating Procedure cut-off value was set as 35 Ct, samples with higher Ct values were considered doubtful, given sigmoidal shape of related amplification plot; otherwise they were considered negative.

### Histopathological examination

From day 2 after IHNV challenge heart sections from 122 fish were assessed by histopathological examination.

Tissue samples stored in 10% neutral buffered formalin were embedded in paraffin and routinely processed into sections of 3–4 µm thickness, stained with haematoxylin and eosin (H&E) and examined by light microscopy. Histopathological changes in the heart consistent with HSMI were scored as none or very sparse (0–0.5), mild focal (1), mild to moderate multifocal (1.5), moderate diffuse (2) and severe diffuse findings (2.5), modified after guidelines previously provided [14] (Figure 5).

### Immunofluorescence antibody test (IFAT) for PRV

An immunofluorescence antibody test (IFAT) was performed on blood smears using a polyclonal antibody raised in rabbits against the putative PRV outer capsid protein ơ1 (anti-ơ1) [23] as described previously [24]. The panel of samples included five selected specimens from PRV-infected blood collected at the peak of viremia, chosen based on low Ct levels. Uninfected blood samples, i.e. negative by RT-qPCR, were used as negative controls.

### Immune gene response analysis

A selection of spleen samples from PRV cohabitants (*n* = 3–6 per sampling point) were homogenized individually in 500 µL QIAzol Lysis Reagent (QIAGEN) with 5 mm steel beads in a TissueLyser II (QIAGEN). Total RNA was isolated by chloroform extraction and ethanol precipitation and loaded onto an RNeasy mini spin column (QIAGEN). Further purification was performed according to the RNeasy kit instructions, and the final RNA concentration was measured using a NanoDropTM 2000 spectrophotometer. Total RNA (500 ng) from each sample was used for cDNA synthesis using the QuantiTect Reverse Transcription kit (QIAGEN) with a genomic DNA elimination step. For RT-qPCR analysis, cDNA corresponding to 10 ng RNA was analysed in triplets for 40 cycles of 94 °C for 15 s and 60 °C for 30 s. Levels of elongation factor 1 α (EF1α), Mx-1, interferon (IFN)a and IFNc mRNA were assessed using 500 nM forward and reverse primers [25], and the Maxima SYBR Green/ROX qPCR Master Mix (Fisher Scientific). The specificity of the SYBR Green assay was confirmed by melting point analysis. An eight point concentration standard curve (two-fold dilutions) made from a representable mix of samples was run on each plate and used to calculate relative gene expression differences. Levels of EF1α mRNA were used for normalization [12].

### Statistics

To compare the differential survival rate, the Kaplan–Meier estimator was used. Log-rank (Mantel-Cox) test was applied to verify the equality of survivor functions across groups. To investigate the difference measured in haematocrit and haemoglobin levels between the different experimental groups One way ANOVA followed by Kruskall–Wallis test was performed. All statistical analyses were performed using GraphPad Prism version 6.00 for Windows, GraphPad Software, La Jolla, California, USA).

## Results

### Protective effect of PRV infection against subsequent IHNV challenge

**Figure 2:**
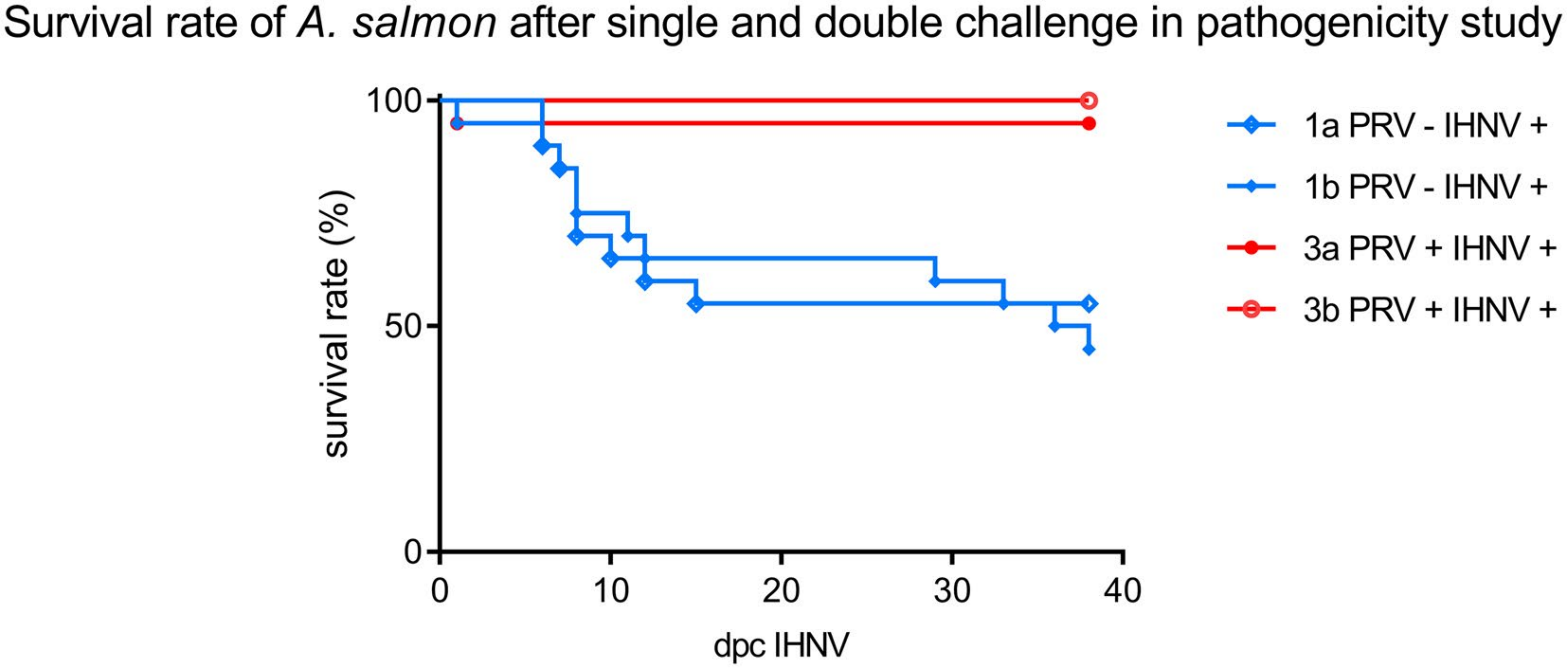
**Survival rate of Atlantic salmon in pathogenicity studies.** Kaplan–Meier survival curves for the PRV−/IHNV+, PRV+/IHNV+ groups describing the pathogenicity study conducted in duplicate 8 L bowls with 20 fish in each unit of the groups.

In the pathogenesis trial conducted in the 150 L tanks 2, 4, 5 and 6, onset of morbidity started as early as 5 days post IHNV challenge and reached 7% at the end of the trial (data now shown). In the pathogenicity trial conducted in the 8 L bowls, morbidity was observed from 6 days post IHNV challenge, apart from one fish which succumbed on day 1 likely due to handling procedures during transfer. Survival rates differed significantly (Log Rank Mantel-Cox test *p* < 0.0001) between the co-challenged group PRV+ IHNV+ (average survival 97.5% SD 2.5%) and PRV− IHNV+ (average survival 50%, SD 5%) (Figure 2). No morbidity was recorded in PRV+ IHNV− tanks, in co-challenged tanks PRV+ IHNV+ and negative control tanks. Clinically affected specimens showed typical IHN signs such as pale gills, skin darkening, exophthalmia and petechial bleedings in internal organs. Presence of IHNV RNA was confirmed by RT-qPCR as the causative agent of morbidity (Alencar et al., in preparation).

### Time course detection of PRV and IHNV

**Figure 3:**
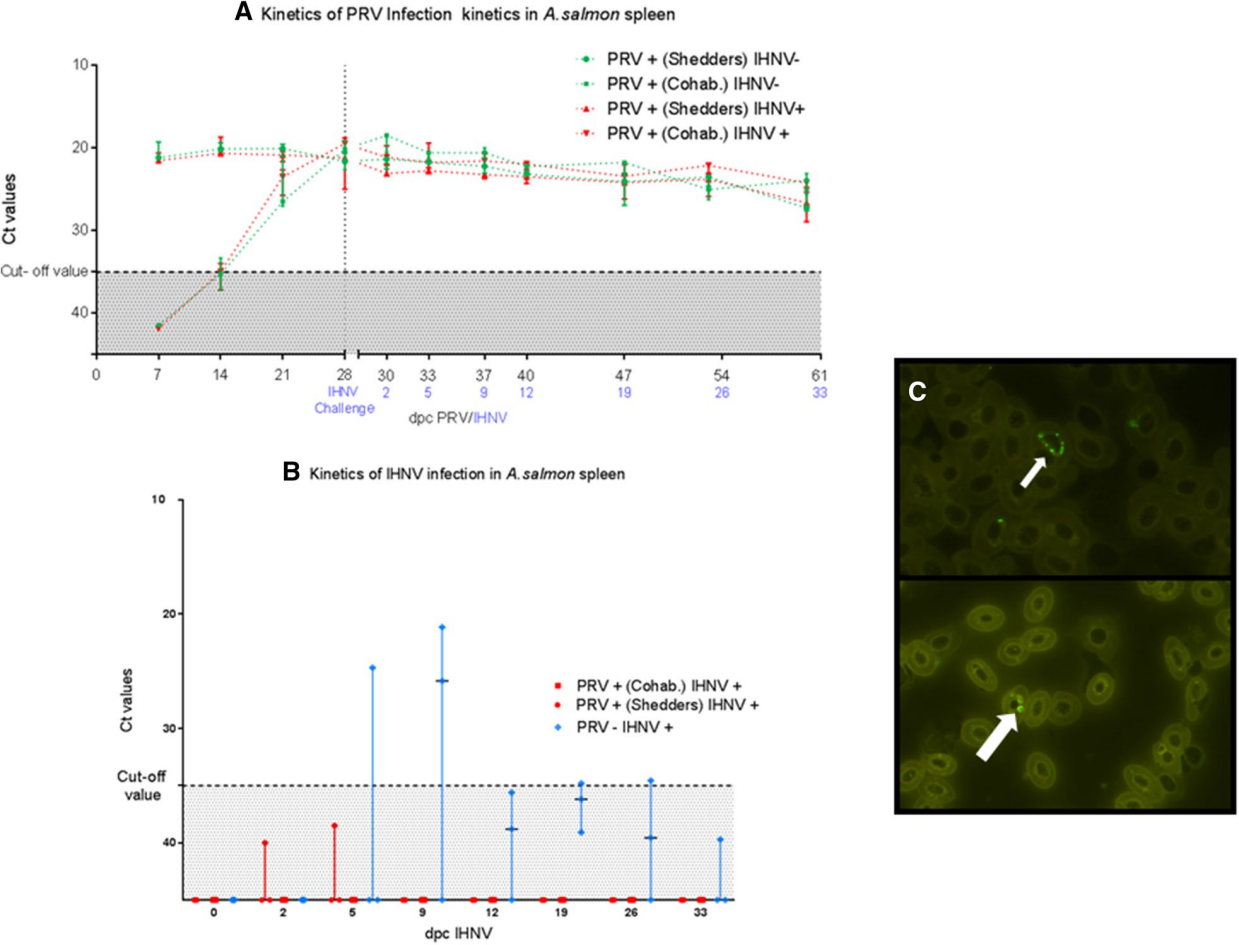
**Infection kinetics of PRV and IHNV in** ***A. salmon.*** **A** PRV established a persistent infection. Challenge with IHNV did not influence PRV relative viral loads measured in spleens. Marks represent the median value of Ct obtained for each group at each time point. **B** IHNV challenge resulted in acute infection peaking 9 days post-challenge followed by a rapid decrease of the viral loads in the spleen. Negligible levels of IHNV infection are observed in PRV previously challenged fish. Horizontal lines represent the median value of Ct obtained from three individuals for each group at each time point. **C** Perinuclear immunofluorescent staining of PRV σ1 antigen in Atlantic salmon erythrocytes infected with PRV (100×).

PRV infection peaked at 4 weeks post challenge in cohabitants, and maintained a persistent plateau phase until the end of the infection trial (Figure 3A). Median Ct value obtained from spleen samples at the maximum level of infection was 19.8 (± 2.2) in cohabitants, and 21.4 (± 0.46) in shedders. At the last sampling 51 days post PRV challenge in PRV+ IHNV−, the median PRV Ct value was 23.9 (± 1.03) in cohabitants and 26.1 (± 1.15) in the shedders. In the co-challenged group, the median Ct value was 24.2 (± 2.2 SD) in cohabitants and 26.6 (± 1.7) in shedders. No significant difference in the Ct values was observed when comparing the groups (Figure 3A).

IHNV infection kinetics developed as an acute infection characterized by high viral loads in spleen followed by substantial decrease in the PRV− IHNV+ group. IHNV RNA was detected in spleen as early as 5 days post IHNV challenge (1 of 3 fish tested IHNV positive, Ct 24.7); and at day 9, 2 of 3 fish tested IHNV positive (Ct 25.8 and 21.1). Thereafter, the Ct values and the number of positive fish decreased and at the last sampling at day 33, IHNV RNA was detected at negligible level in only 1 sample (Ct 39.7).

In the co-challenged group PRV+ IHNV+, IHNV RNA was detected at negligible levels in only 1 sample throughout the whole experiment, at day 5 post IHNV challenge (Ct 38.5) (Figure 3B).

The results obtained by RT-qPCR were corroborated corroborated by immunofluorescent detection of PRV outer capsid protein σ1 in cytoplasmic and perinuclear inclusions of blood cells from (Figure 3C).

### Modulation of IFNa, IFNc and Mx expression in PRV infected and IHNV co-challenged fish

**Figure 4:**
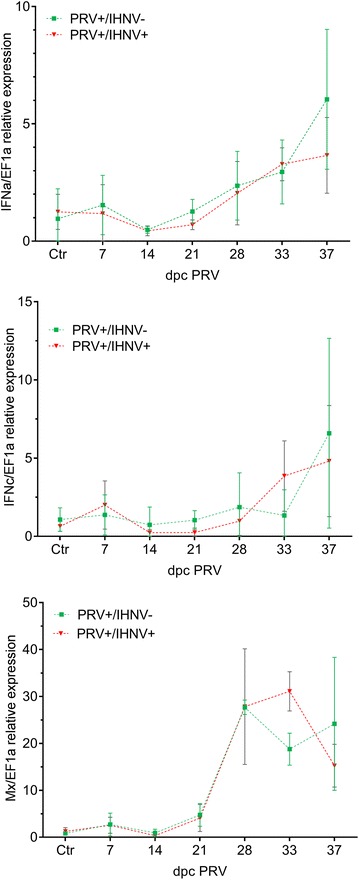
**Expression of IFNa, IFNc and Mx genes in spleen during PRV infection and effect of IHNV co-challenge.** Fold increase in gene expression of interferon (IFN)a (**A**), IFNc (**B**) and the IFN− regulated antiviral gene Mx (**C**) in spleen during the course of infection with PRV in Atlantic salmon cohabitants from tank 4 (PRV+/IHNV+) and tank 5 (PRV+/IHNV−). Gene expression is normalized to the reference gene EF1α, and shown as fold induction compared to uninfected controls from tank 6 (PRV−/IHNV−).

Fish challenged with PRV by cohabitation were analysed for expression of the antiviral genes IFNa, IFNc and Mx in spleen (Figure 4). A mean two-fold increase in IFNa and a 30 fold increase in Mx expression relative to expression levels in uninfected fish was observed at 4 weeks post PRV challenge, at the time when the fish were co-challenged with IHNV. After this, the IFN expression continued to increase up to five to sixfold in PRV+/IHNV− fish whereas Mx slightly decreased (Figure 4). In comparison, the co-challenged fish PRV+/IHNV+ tended to have lower expression of IFN (both a and c) and Mx 9 days after IHNV co-infection compared to PRV+/IHNV− although these results were not statistically significant.

### HSMI prevalence and severity

**Figure 5:**
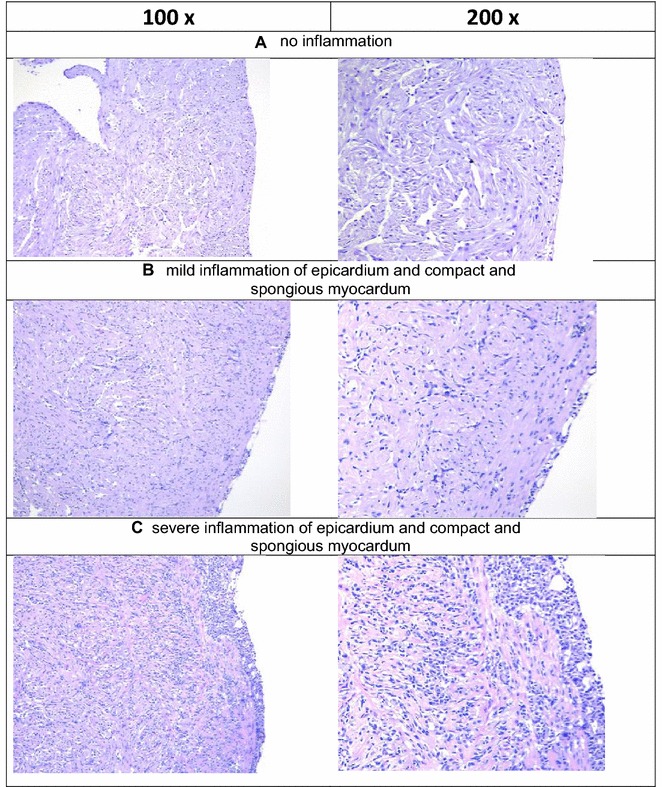
**Grading of histopathological changes related to development of HSMI in** ***A. salmon*** **heart.** H&E stained slides. **A** No inflammation observed corresponding to grade 0 from (PRV+/IHNV+) co-challenged cohabitant 2 days post-challenge (dpc). IHNV. **B** Mild inflammation of epicardium and compact and *spongiousus* layer of myocardium corresponding to grade 1 from PRV+/IHNV− challenged shedder 26 dpc IHNV. **C** severe inflammation of epicardium and compact and *spongiousus* layer of myocardium corresponding to grade “2.5” from PRV+/IHNV− challenged cohabitant 19 dpc IHNV.

The histopathological investigation revealed heart lesions consistent with HSMI in both the PRV+/IHNV− and PRV+/IHNV+ groups with a large proportion of hearts affected (78 and 76% respectively) (Table 1, Figure 5 and Additional file 1). The extent of lesions varied from mild (Figure 5B) to severe (Figure 5C). From day 12 post IHNV challenge, six of the seven hearts (86%) with the most severe findings were in the PRV+/IHNV− cohabitants group. Comparatively, 89% of the PRV+ IHNV+ cohabitants fish sampled during the same period showed mild to moderate inflammation (Figure 5 and Additional file 1). Fish examined in the PRV−/IHNV+ and negative control groups had no heart pathology (Figure 5A).

### Variations in hematocrit and hemoglobin during infection

**Figure 6:**
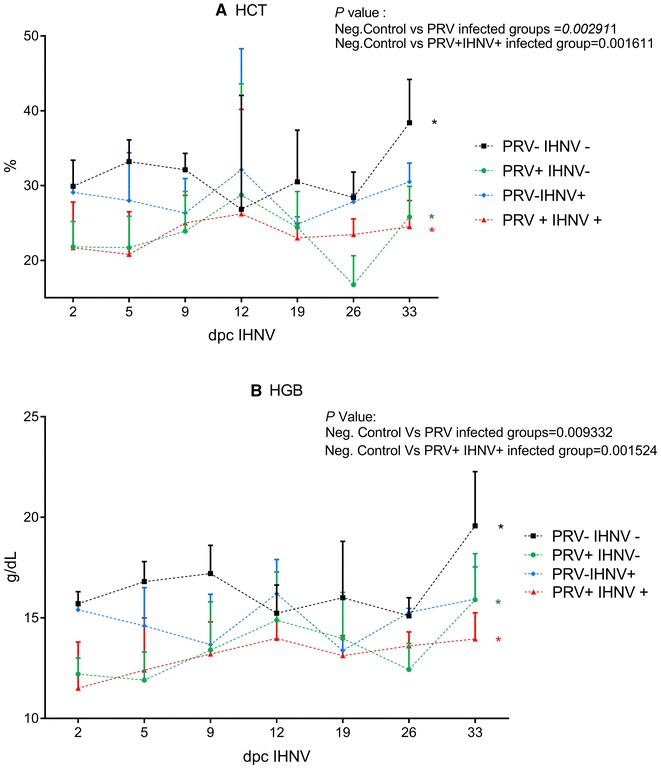
**Haematocrit and haemoglobin levels are reduced by PRV and IHNV infection.** Haematocrit levels (**A**) and haemoglobin levels (**B**) monitored in experimental groups (PRV−/IHNV−, PRV+/IHNV−, PRV−/IHNV+, PRV+/IHNV+) during pathogenesis trial. Mean level and SD from the different groups at selected time points are indicated by vertical lines.

Hematocrit (Hct) and hemoglobin (Hgb) measurements revealed significant differences (Kruskall–Wallis test Hct *p* < 0.002 and Hgb *p* < 0.05) when comparing PRV+/IHNV− and co-challenged PRV+/IHNV+ groups to the negative control group PRV−/IHNV− (Figure 6).

## Discussion

Salmonid aquaculture worldwide largely relies on production in net pens. This production mode introduces a risk of potential transfer of pathogens between wild and farmed stocks. Wild stocks may harbor several infective agents, and farmed fish may act as amplifiers of pathogens posing a risk to the wild stocks in the eco-system of the farming area. Therefore, knowledge about host–pathogen interactions and pathogen–pathogen interplay is critical for both ecological and economic reasons, and thus for the development of a sustainable salmon farming industry.

This study brings interesting elements to consider for an exhaustive risk assessment analysis of the spread of IHNV from continental Europe to Atlantic salmon aquaculture industry in northern Europe. The recent report from November 2017 (OIE notification) of IHN outbreaks in rainbow trout in Finland highlights the importance of such an assessment.

The specific aim was to investigate the effect of PRV infection, which is very common in Atlantic salmon in Northern Europe, to a subsequent exposure to IHNV, belonging to the European genogroup E.

The results showed significant IHNV resistance in PRV infected compared to non-PRV infected Atlantic salmon. The PRV infected, IHNV challenged fish showed no mortality and only negligible levels of IHNV RNA were detected. On the contrary, Atlantic salmon not exposed to PRV showed clinical signs of IHN such as skin darkening, exophthalmia and lethargy, leading to reduced survival rate of 93% in 150 L tanks and 50% in 8 L bowls. The difference between the survival rates in the two settings can be related to the higher biomass and density in this experimental unit (13 kg/m^3^ in tanks and 70 kg/m^3^ in bowls), as it has been previously reported that host density is a key factor for the incidence and severity of IHNV infections [26].

Our findings are in agreement with the results obtained by Lund et al. [19] where it was demonstrated that a preceding PRV infection mediated protection against a subsequent SAV challenge, shown by reduction in SAV RNA levels and reduced severity of the related pathological lesions. On the other hand, our findings diverge considerably from those described by Polinski et al. [27], where a PRV challenge conducted in Sockeye salmon (*Oncorhynchus nerka*) using a viral strain from British Columbia showed no effect on host immune responses or protection against a subsequent IHNV infection. However, some significant differences in the two experimental settings may provide explanations for the differences observed. Firstly, different species were infected in the trials; *S. salar* (this study) and *O. nerka* in the Polinsky study, and different species often show different responses to the same pathogen [28, 29]. One example of this is that the PRV strain found in rainbow trout in Europe cause an HSMI like disease in this species, but only affects Atlantic salmon to a minor degree and with little induction of antiviral responses [24]. Secondly, the PRV challenges were performed differently, since i.p. injection of virus was performed in the Sockeye salmon challenge, while a cohabitation trial, which better mimics natural infection, was used in our Atlantic salmon challenge. It is also worth reporting that the peak viral load was slightly higher in the cohabitant fish than in the i.p. injected shedders in our experiment. Thirdly, the Sockeye salmon were subjected to IHNV immersion challenge 2 weeks after PRV injection, while in our study PRV infection were allowed to develop for 4 weeks, reaching peak viral loads prior to IHNV immersion challenge. Notably, according to Dahle et al. [15], the peak in PRV viral load is correlated with the peak induction of IFN-regulated antiviral responses in Atlantic salmon blood cells. Finally, different viral strains were included in the different studies. We used a Norwegian PRV strain shown to cause HSMI, whereas the ability of the British Columbia PRV strain to cause disease is not clearly defined yet [8, 30]. In regard to IHNV, a European isolate was used in this study as part of a targeted risk assessment, whereas the American isolate BC93-057, genogroup U was used in the study of Polinski [27].

The difference between the IHNV susceptibility of the PRV+/IHNV+ group and the PRV−/IHNV+ group was striking. The PRV−/IHNV+ showed 50% survival while in the co-challenged group all fish survived. This suggests that PRV infection is highly protective against the IHNV E genogroup, which is well described in terms of phenotypic [20] and genetic profile [31]. This finding was corroborated by RT-qPCR analysis assessing the presence of PRV and IHNV genome in spleen samples throughout the experiment. IHNV was detected in most of the non-PRV infected fish, depicting the kinetics of an acute infection, while IHNV was detected at a very low level (Ct 38.5) in only one of the PRV-infected fish.

In agreement with previous studies performed in Atlantic salmon [15, 32] PRV induced an increase in the gene expression of IFN (IFNa and IFNc) and Mx. In general, this immunological pathway is pivotal in protection against viral infections in vertebrates, including Atlantic salmon [33, 34]. In our experiment, PRV induced increased IFNa and Mx expression in spleen, the organ where PRV-infected erythrocytes are likely to accumulate.

In rainbow trout, effective protection from virulent novirhabdoviruses is shown to be elicited through the IFN response, as indicated by the protective effect of a preceding infection with virus such as IPNV, which, like PRV, is a non-enveloped dsRNA virus [35, 36]. IFN regulated genes are also induced by intramuscular administration of DNA vaccines against IHNV [37]. DNA vaccination against IHNV has proven to give effective protection already as early as 4 days after injection against homologous [38] and heterologous novirhabdoviruses [39], highlighting the importance of innate immune responses in protection against novirhabdovirus.

In order to assess the pathological effect of viral challenge, heart, which is an important target organ for the two pathogens, was analysed by histology. The majority of the hearts from fish in both PRV+/IHNV− and PRV+/IHNV+ groups developed histopathological findings consistent with HSMI. The lesions varied from mild to severe, and there was no difference in the prevalence of heart lesions between the two groups. However, an interesting trend was observed when scoring the severity of the lesions in the two groups, as six out of seven hearts with the most severe findings were seen in the hearts of PRV+/IHNV− cohabitants group, suggesting that exposure to IHNV may possibly modify the host inflammatory response to the PRV infection. This observation however has to be further investigated.

Interestingly, both PRV and IHNV infection significantly affected haematocrit and haemoglobin levels. PRV infected fish suffered from significant anaemia (as measured by both parameters) compared to negative control groups at the time of IHNV exposure in the trial, which corresponds to the peak in PRV load. Similarly, in the PRV−/IHNV+ group, the haemoglobin parameter was significantly reduced compared to the control group at the end of the experiment, but with largest reduction at day 9 post IHNV exposure, i.e. correlating with the peak of IHNV infection.

In conclusion, the results show that Atlantic salmon is susceptible to the IHNV genogroup E, which is present in continental Europe, and that a preceding PRV infection protects against subsequent IHNV challenge when conducted at the peak of PRV viremia. The protection observed in this study is likely to be related to the PRV-induced innate antiviral responses, such as IFN and IFN-stimulated genes. Further studies are needed to unravel the mechanism behind the observed protection, the duration of protection, and its possible specificity towards other viral pathogens.

## Additional file

**Additional file 1.** **Grading of histopathological changes related to development of HSMI in A. salmon heart.** This table displays histopathological grading of HSMI lesions in heart sections of A. salmon H&E stained. Median value per time point of each group is calculated.
