# Supplementary material for: Piscine orthoreovirus infection in Atlantic salmon (Salmo salar) protects against subsequent challenge with infectious hematopoietic necrosis virus (IHNV)
Source: Vet Res. 2018 Mar 13;49:30. doi: 10.1186/s13567-018-0524-z (PMC5850924; doi:10.1186/s13567-018-0524-z)
Supplement: Supplementary file 1 — Additional file 1. Grading of histopathological changes related to development of HSMI in A. salmon heart. This table displays histopathological grading of HSMI lesions in heart sections of A. salmon H&E stained. Median value per time point of each group is calculated. [file 13567_2018_524_MOESM1_ESM.docx]

**Additional file 1 Grading of histopathological changes related to development of HSMI in *A. salmon* heart.**

|  | **Dpc IHNV** | **2** | | | **5** | | **9** | | **12** | | **19** | | **26** | | **33** | |
| --- | --- | --- | --- | --- | --- | --- | --- | --- | --- | --- | --- | --- | --- | --- | --- | --- |
| **PRV- IHNV-** |  | | nd | nd | | nd | | nd | | 0 | | 0 | | 0 | |  |
|  |  | | 0 | 0 | | 0 | | 0 | | 0 | | 0 | | 0 | |  |
|  |  | | 0 | 0 | | 0 | | 0 | | 0 | | 0 | | 0 | |  |
|  | **Median** | **n/a** | | | **n/a** | | **n/a** | | **n/a** | | **n/a** | | **n/a** | | **n/a** | |
| **PRV+ (Shedd.) IHNV -** |  | | 1,5 | 2 | | 2 | | 1,5 | | 1 | | 1 | | 0 | |  |
|  |  | | nd | 1,5 | | 1,5 | | 1,5 | | 2 | | 1 | | 1 | |  |
|  |  | | 0 | 1,5 | | 1,5 | | 0 | | 1 | | 1,5 | | 0 | |  |
|  | **Median** | **0** | | | **1,5** | | **1,5** | | **1,5** | | **1** | | **1** | | **n/a** | |
| **PRV + (Shedd.) IHNV+** |  | | 1 | 1 | | 1,5 | | 1,5 | | 0 | | 1,5 | | 0 | |  |
|  |  | | 0 | 1 | | 1,5 | | 1 | | 1 | | 1 | | 1,5 | |  |
|  |  | | 0 | 1 | | 1 | | 1 | | 1 | | 1 | | 0 | |  |
|  | **Median** | **n/a** | | | **1** | | **1,5** | | **1** | | **1** | | **1** | | **n/a** | |
| **PRV + (Cohab.) IHNV -** |  | | 0 | 0 | | 1 | | 1,5 | | 2,5 | | 2,5 | | 1,5 | |  |
|  |  | | 0 | 1 | | 1 | | 1,5 | | 2,5 | | 2,5 | | 2,5 | |  |
|  |  | | 0 | 0 | | 1 | | 1,5 | | 1 | | 2 | | 2,5 | |  |
|  | **Median** | **n/a** | | | **n/a** | | **1** | | **1,5** | | **2,5** | | **2,5** | | **2,5** | |
| **PRV + (Cohab.) IHNV +** |  | | 0 | 0 | | 1,5 | | 2,5 | | 1,5 | | 1,5 | | 2 | |  |
|  |  | | 0 | 0 | | 1,5 | | 2 | | 1,5 | | 1,5 | | 0 | |  |
|  |  | | 0 | 0 | | 1 | | 1,5 | | 1,5 | | 2 | | 2 | |  |
|  | **Median** | **n/a** | | | **n/a** | | **1,5** | | **2** | | **1,5** | | **1,5** | | **2** | |
| **PRV - IHNV +** |  | | 0 | 0 | | 0 | | 0 | | 0 | | 0 | | 0 | |  |
|  |  | | 0 | 0 | | 0 | | 0 | | 0 | | 0 | | 0 | |  |
|  |  | | 0 | 0 | | 0 | | 0 | | 0 | | 0 | | 0 | |  |
|  | **Median** | **n/a** | | | **n/a** | | **n/a** | | **n/a** | | **n/a** | | **n/a** | | **n/a** | |

Histopathological grading of HSMI lesions in heart sections of *A.salmon* H&E stained. Median value per time point of each group is calculated.
